# Spatial frequency analysis detects altered tissue organization following hamstring strain injury at time of injury but not return to sport

**DOI:** 10.1186/s12880-021-00721-1

**Published:** 2021-12-10

**Authors:** Scott K. Crawford, Christa M. Wille, Mikel R. Stiffler-Joachim, Kenneth S. Lee, Greg R. Bashford, Bryan C. Heiderscheit

**Affiliations:** 1 Department of Kinesiology, University of Wisconsin-Madison, 1300 University Ave, Madison, WI 53706 USA; 2 Department of Orthopedics and Rehabilitation, University of Wisconsin-Madison, Madison, WI USA; 3 Department of Biomedical Engineering, University of Wisconsin-Madison, Madison, WI USA; 4 Badger Athletic Performance Program, University of Wisconsin-Madison, Madison, WI USA; 5 Department of Radiology, University of Wisconsin-Madison, Madison, WI USA; 6 Department of Biological Systems Engineering, University of Nebraska, Lincoln, NE USA

**Keywords:** Ultrasound, Magnetic resonance imaging, Hamstring, Injury, Return to sport, Athlete

## Abstract

**Background:**

Hamstring strain injury (HSI) diagnosis is often corroborated using ultrasound. Spatial frequency analysis (SFA) is a quantitative ultrasound method that has proven useful in characterizing altered tissue organization. The purpose of this study was to determine changes in muscular tissue organization using SFA following HSI.

**Methods:**

Ultrasound B-mode images were captured at time of injury (TOI) and return to sport (RTS) in collegiate athletes who sustained an HSI. Spatial frequency parameters extracted from two-dimensional Fourier Transforms in user-defined regions of interest (ROI) were analyzed. Separate ROIs encompassed injured and adjacent tissue within the same image of the injured limb and mirrored locations in the contralateral limb at TOI. The ROIs for RTS images were drawn to correspond to the injury-matched location determined from TOI imaging. Peak spatial frequency radius (PSFR) and the fascicular banded pattern relative to image background (Mmax%) were compared between injured and adjacent portions within the same image with separate paired t-tests. Within-image differences of SFA parameters in the injured limb were calculated and compared between TOI and RTS with Wilcoxon rank sum tests.

**Results:**

Within the injured limb at TOI, PSFR differences in injured and healthy regions did not strictly meet statistical significance (*p* = 0.06), while Mmax% was different between regions (*p* < 0.001). No differences were observed between regions in the contralateral limb at TOI (PSFR, *p* = 0.16; Mmax%, *p* = 0.30). Significant within-image differences in PSFR (*p* = 0.03) and Mmax% (*p* = 0.04) at RTS were detected relative to TOI.

**Conclusions:**

These findings are a first step in determining the usefulness of SFA in muscle injury characterization and provide quantitative assessment of both fascicular disruption and edema presence in acute HSI.

## Introduction

Clinical diagnosis of hamstring strain injury (HSI) is often aided with medical imaging modalities such as magnetic resonance imaging (MRI) or ultrasound (US). In fact, many HSI grading systems integrate findings from medical imaging to classify injury severity, such as fiber disruption and changes in signal intensity [1–3]. However, the role of MRI or US in HSI diagnosis has been limited with even more ambiguity related to their prognostic value in determining an athlete’s ability to return to play [4, 5]. MRI is considered the ‘gold-standard’ for soft tissue imaging due to its high contrast resolution and its ability to distinguish soft tissue anatomy. Yet, moderate agreement in HSI identification between MRI and US has been observed [6]. Given that MRI is expensive and infeasible in some clinical settings while US is portable and less expensive, US may be a useful auxiliary diagnostic imaging tool [7]. Current US methodologies indicate injury primarily on qualitative determination of normal versus abnormal echogenic appearance and presence of edema [6, 8] or by measuring the length of the abnormal hypoechoic region [4], which may not provide meaningful insight into fiber disruption or prognostic value of US imaging in acute HSI [4, 9].

The advent of better US systems, high frequency transducers, and faster computational speeds has led to increased utilization of musculoskeletal US and quantitative US methods. Some studies have quantified tissue health using first order gray-level statistics of echogenicity by reporting mean or median pixel echointensity [10–12]. Although these metrics have been correlated to muscle quality, strength, and fatty infiltration [10, 11, 13, 14], they do not provide insight into the overall structural organization of the tissue—such as the repeated perimysial bundle pattern—especially with respect to muscle strain injury.

In contrast, spatial frequency analysis (SFA) is a quantitative US method that characterizes tissue structure and organization from the two-dimensional frequency distribution of the coherent speckle striation patterns observed in B-mode images. Parameters extracted from these spatial frequency distributions have been correlated with the organization and mechanical properties of collagen fascicles in tendon [15, 16]. Specifically, the peak spatial frequency radius (PSFR), which characterizes the spacing between collagen bundles, has proved useful in differentiating between healthy and tendinopathic tissue [15, 17]. In general terms, a more compact fascicular striation pattern results in a higher dominant spatial frequency—and subsequently a higher PSFR value—indicating healthy tendon [15, 18]. Another parameter that might prove useful in characterizing tissue organization is Mmax% [15, 19], which represents the strength of the most prominent hyperechoic striation pattern reflected by the fascicles relative to the overall image brightness [20]. Although SFA parameters were originally applied to tendon tissue, recent investigations have adapted SFA for use in the hamstring muscles of healthy individuals and shown it to be reliable [20] and to reflect known architectural differences [18]. However, no study has investigated how SFA parameters associated with the organizational structure of the muscle might change following acute HSI.

Therefore, the purpose of this study was to use SFA to characterize changes in muscle tissue organization following HSI at time of injury (TOI) and return to sport (RTS) in a cross-sectional design. The first aim of the study was to investigate if SFA parameters differed between the injured portion of the muscle relative to a healthy portion of the same muscle at TOI. Additionally, we investigated if mirrored injured and healthy regions from the injured limb differed within the homologous muscle of the contralateral limb. We hypothesized that SFA would be able to differentiate between areas of injury compared to healthy tissue and that PSFR and Mmax% values would be different between the injured and healthy regions of the involved limb based upon observations in tendon [15, 17]. Regarding the contralateral limb, we hypothesized there would be no difference in SFA parameters between healthy and injury-matched locations. The second aim of the study was to investigate differences between the regions of injury and healthy muscle between TOI and RTS on the involved limb only. Large differences in SFA parameters between injured and healthy muscle would be expected in an acute injury but likely diminish over time as the tissue heals. Thus, we hypothesized that within-image differences in PSFR and Mmax% between heathy and injured portions of the involved limb would be different between TOI and RTS.

## Materials and methods

The data presented in this study were collected as part of a larger observational, prospective investigation of collegiate athletes who sustained an HSI. The Health Sciences Institutional Review Board at the University of Wisconsin-Madison approved all procedures. Athletes provided informed consent prior to participation. Male and female collegiate athletes who participated in either American football, soccer, or track and field and sought medical attention for an HSI within 7 days after the initial injury mechanism were recruited to participate in the study. An HSI was defined as sudden onset of pain in the posterior thigh that occurred during sport participation, which required medical attention, and resulted in the athlete losing one or more days from training or competition. Injuries were confirmed by the athlete’s medical team. Injury determination was made by the athlete’s medical team based upon the presence of two or more of the following symptoms: palpable pain along any of the hamstring muscles, posterior thigh pain without radicular symptoms during straight leg raise testing, weakness with resisted knee flexion, pain with resisted knee flexion, and posterior thigh pain with sports/running.

T2-weighted MRI and longitudinal B-mode US imaging were obtained within 7 days of sustaining the HSI [21]. A standardized rehabilitation protocol was implemented by the team’s athletic trainer and RTS was determined when medical clearance was obtained to resume all sport-related activities [22]. General criteria for RTS clearance included: absence of hamstring-specific pain upon palpation; symmetry between limbs for manually-resisted isometric strength and range of motion; and absence of pain or stiffness during high speed running [22]. MRI and US imaging were repeated within 7 days of RTS. If imaging could not be obtained within one week of a time point, then that time point was noted as missed.

**Fig. 1:**
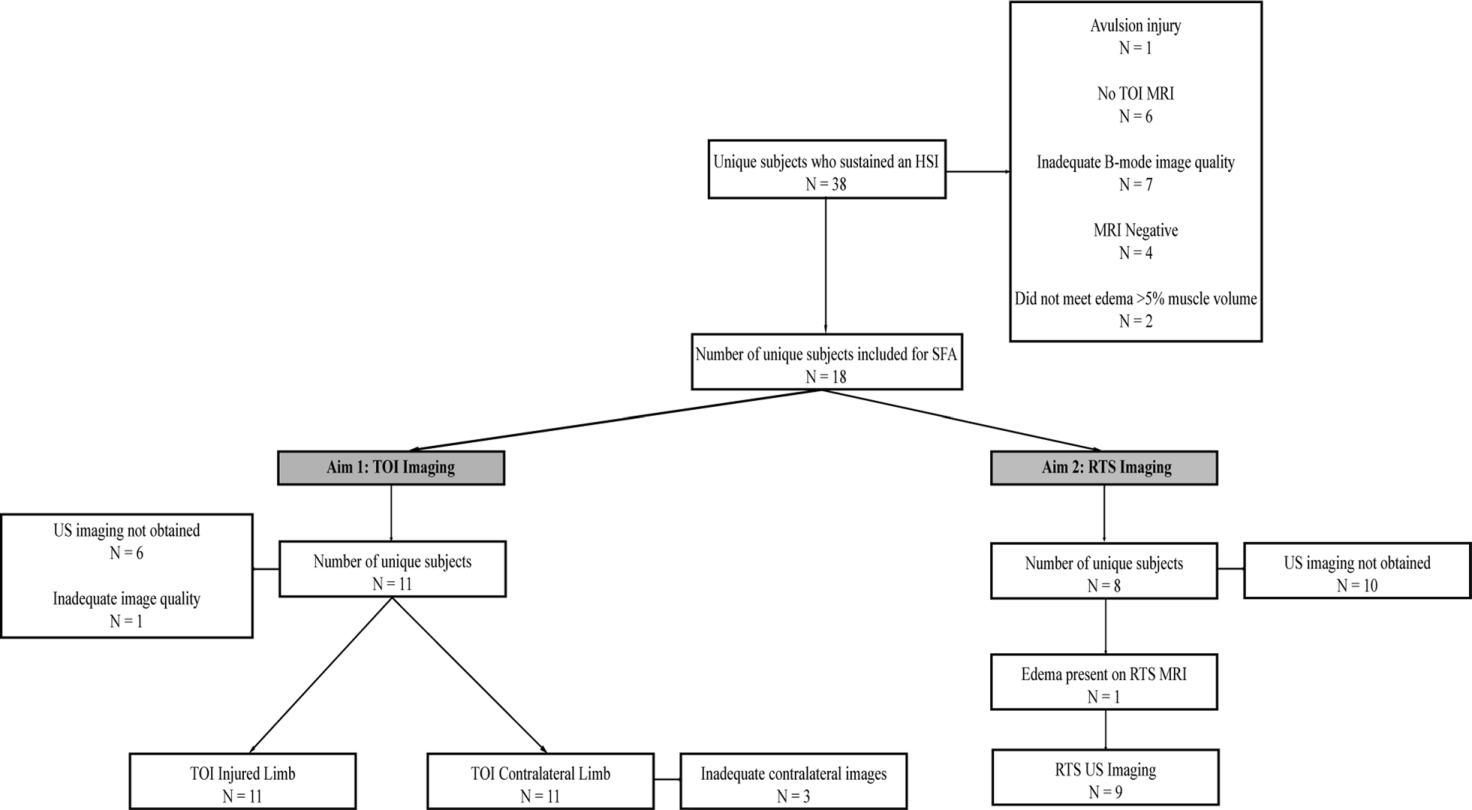
Flowchart of athletes included for spatial frequency analysis of ultrasound B-mode images at time of injury and return to sport. *HSI* hamstring strain injury, *MRI* magnetic resonance imaging, *RTS* return to sport, *US* ultrasound, *TOI* time of injury

Data from the larger project were included in this analysis if an athlete: (1) had a visible injury on TOI MRI with edema volume greater than 5% of the total muscle volume; (2) had US images with no image saturation at TOI or RTS; and (3) did not incur a complete tear or avulsion injury (Fig. 1). Only athletes with injuries visible on MRI were included to ensure proper injury locations were identified. The presence and location of injury on MRI were identified as a region of hyperintense signal representing edema, confirmed by an experienced musculoskeletal radiologist. If the athlete had an MRI at TOI but only had US imaging at RTS, the injury location was determined from the location of edema in the MRI and classified as either superficial or deep. In one athlete included in the analysis, no TOI MRI was performed but injury and edema were still noted in the RTS MRI; therefore, the injury location identified from the RTS MRI was used.

### Imaging procedures

Participants received a bilateral MRI examination of the thighs. A 3.0 T scanner (MR750 GE Healthcare Discovery, Waukesha, WI) was used with a 32-channel full torso coil with the athlete positioned feet first into the scanner. Axial and coronal views were acquired using a T1-weighted sequence (TR/TE = 750/16 ms, 40 cm field of view, 480 × 480 matrix, 3 mm slice thickness) for assessment of anatomy and a fat-suppressed T2-weighted sequence (TR/TE = 4400/70 ms, 40 cm field of view, 512 × 512 matrix, 3 mm slice thickness) was used for assessment of muscle edema.

Clinical US imaging was performed by a trained musculoskeletal sonographer using a commercial US system (Aixplorer, Supersonic Imagine, Weston, FL) with a high-frequency linear array transducer (SuperLinear SL10-2, 38 mm aperture). Two sonographers (both with over 5 years of musculoskeletal US experience) were used throughout the course of the study due to staff turnover; however, both sonographers were informed of the study design and standardized image acquisition protocol prior to data collection and were supervised by the same musculoskeletal radiologist with 18 years of experience. The injury site was defined as the site of maximum tenderness to palpation. A transverse view of the muscle was first visualized to ensure the involved muscle was imaged. Longitudinal B-mode images were acquired at the injury site on the involved limb and on the contralateral limb. The location of the injury site was measured with respect to the ischial tuberosity and recorded. This location was matched on the contralateral uninjured limb.

### Ultrasound B-mode image analysis

**Fig. 2:**
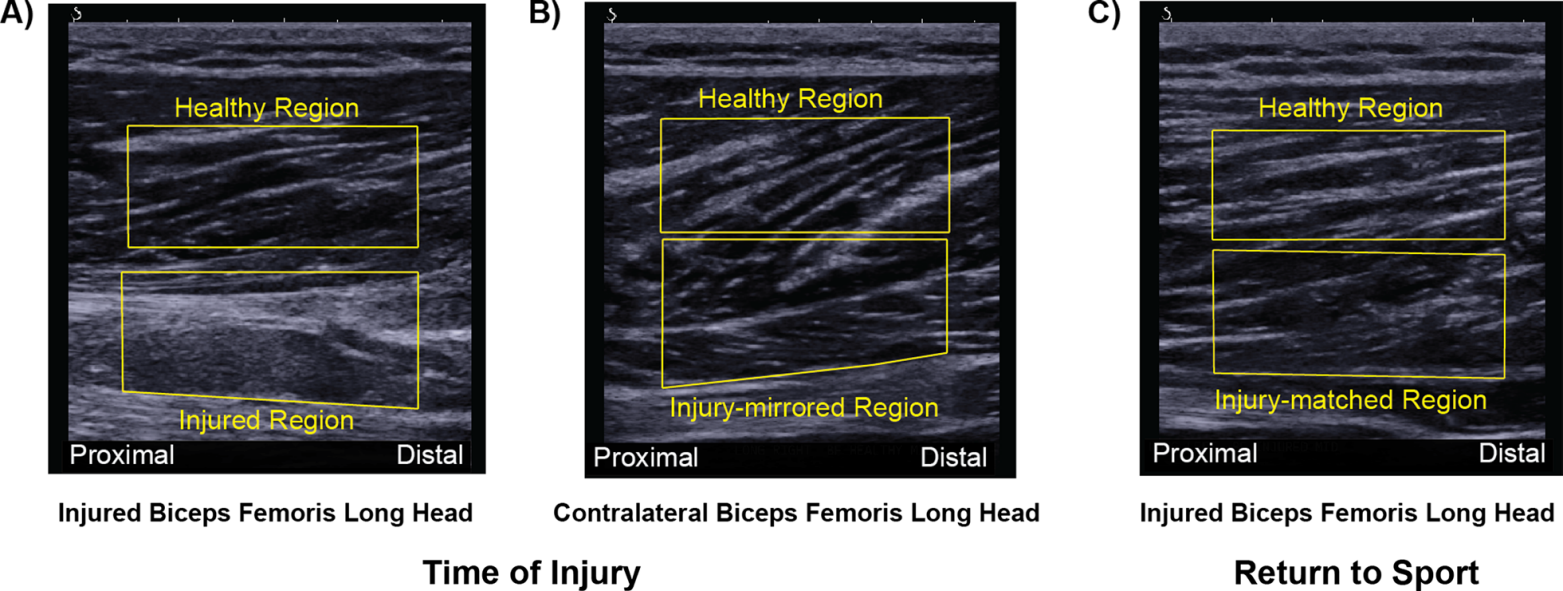
Representative B-mode images from one athlete with regions of interest (ROI) drawn. **A** Injured biceps femoris long head (BFlh) at time of injury. An ROI was drawn about the region with visually disrupted echotexture or increased echointensity (injured). A second ROI was drawn to capture tissue adjacent to the injured ROI without overlap of the two ROIs (healthy). **B** Contralateral BFlh at time of injury. The ROIs were drawn to mirror those from the injured BFlh. **C** Injured BFlh at return to sport (RTS). The ROI was drawn to match those from images obtained at time of injury or to correspond with edema noted on MRI at time of injury if no B-mode images were collected within 7 days of injury

Due to different image acquisition parameters and the unknown effects these machine settings may have on subsequent SFA parameter extraction, a within-image analysis was performed where a polygonal region of interest (ROI) was manually drawn about the injured and uninjured (healthy) adjacent portions of muscle within the same image (Fig. 2A). The injured region was defined by the presence of visually disrupted echotexture or increased echointensity [23, 24]. The ROI for the healthy portion was drawn to capture muscle adjacent to the injured ROI without overlap of the two ROIs. For the contralateral limb, the ROIs were manually drawn to mirror the locations of the injured limb (Fig. 2B). A single rater (S.K.C.) drew the ROIs for all images. Excellent reliability in extracted SFA parameters has been shown when a single rater draws the ROIs [20]. The ROIs for RTS US images were drawn to correspond to the injury location determined from either TOI US or MRI as described previously and defined as the injury-matched ROI location for subsequent analyses (Fig. 2C).

The static longitudinal B-mode images were analyzed using custom MATLAB (Mathworks, Natick, MA) algorithms similar to previous studies [15, 20] such that all possible sub-images (kernels) within the ROI were analyzed in the spatial frequency domain. Since imaging depth was optimized for image acquisition, the size of the kernels (in pixels) was calibrated to correspond to a 6.5 × 6.5 mm square, which is consistent with previous investigations [20]. Two-dimensional Fourier transforms were applied within each kernel after zero-padding to 128 × 128 samples to increase frequency sampling. A 2D high-pass filter with a − 3 dB cut-off was applied in the frequency domain to attenuate low spatial frequency artifacts.

The PSFR and Mmax% parameters were extracted from each kernel and averaged across the entire ROI [15]. Within the frequency domain, the PSFR is defined as the distance from the origin to the location of the maximum frequency amplitude and corresponds to the dominant spacing between the perimysium [18]. The Mmax% parameter is the ratio of the amplitude corresponding to the peak spatial frequency and the overall image brightness and indicates the strength of the most dominant banded pattern relative to the background [18]. Pilot data (not shown) exhibited minimal variation in PSFR and Mmax% parameters with small modifications in machine settings typically manipulated in musculoskeletal imaging, whereas other parameters previously reported in muscle [20] vary substantially with changing machine system settings [25].

### Statistical analysis

#### Aim 1: time of injury analysis of injured and contralateral limbs

Comparisons between the extracted parameter values (PSFR and Mmax%) of the injured and adjacent portions were made with separate, paired two-sided t-tests within both the injured and contralateral limb at TOI. Normality of the data was confirmed by visually reviewing Q-Q plots of each parameter at each time point and using the Anderson–Darling test.

#### Aim 2: within-image injured vs. healthy differences between TOI & RTS in injured limb

To evaluate differences between time points, the within-image difference was calculated at both TOI and RTS between the injured and healthy tissue in the image of the injured limb as $Healthy-Injured$, where positive values indicated that the adjacent tissue had a larger PSFR or Mmax% value than the injured portion. Due to non-normal distributions and different participant groups at TOI at RTS, separate unpaired, two-sided Wilcoxon ranked sum tests for PSFR and Mmax% were performed to compare the within-image differences of each parameter between TOI and RTS of the injured limb only. All analyses across both aims were performed in RStudio (Version 1.2.5033, RStudio, Inc.) with a priori significance set to α = 0.05. Data are reported as mean (standard deviation) or as median [interquartile range] as appropriate.

## Results

After accounting for various exclusion criteria (Fig. 1), a total of 11 athletes from American football and track and field were included for image analysis at TOI (Table 1). The mean number of days between HSI occurrence and TOI US imaging was 3.8 (1.8) days. The PSFR of the injured portion (0.77 (0.25) mm^−1^) of the tissue was not significantly different than that of the healthy portion (0.89 (0.24) mm^−1^, *p* = 0.06). Mmax% was significantly lower in the injured region (1.41 (0.43)%) compared to the healthy, adjacent portion of the tissue on the injured limb (2.28 (0.50), *p* < 0.001) (Table 2). No differences were observed between regions in the contralateral limb for either PSFR (0.92 (0.26) vs. 0.83 (0.18) mm^−1^, *p* = 0.16) or Mmax% (2.33 (0.67) vs. 2.49 (0.58)%, *p* = 0.30).

**Table 1:** Demographic information for athletes included for time of injury and return to sport ultrasound imaging

	Time of injury cohort	Return to sport cohort
Number of athletes	11^‡^	9^§^
Age (years)	19.7 (1.4)	20.2 (1.3)
Sex (Female/Male)	0/11	2/7
Height (m)	1.83 (0.05)	1.79 (0.10)
Weight (kg)	86.6 (18.2)	78.5 (16.7)
Body Mass Index (kg/m^2^)	25.7 (4.1)	24.3 (2.9)
Sport (football/track & field)	4/7	2/6
Days between injury/return to sport and imaging (days)	3.8 (1.8)	3.4 (2.3)
Days away from sport	NA^¶^	39.8 (23.7)

^†^Data are expressed as means (standard deviation) or N

^‡^N = 8 for contralateral limb at time of injury

^§^N = 2 athletes included across both TOI and RTS imaging

^¶^NA = not applicable

**Table 2:** Time of injury within-image spatial frequency analysis parameter measures of injured and contralateral limbs

SFA parameter	Imaged limb	Within-image region	Parameter value^†^	*P*-value^‡^ [95% Confidence Interval]
PSFR (mm^−1^)	Involved	Injury site	0.77 (0.24)	*p* = 0.06 [− 0.01, 0.25]
		Adjacent	0.89 (0.25)	
	Contralateral	Mirrored injury site	0.83 (0.18)	*p* = 0.16 [− 0.04, 0.22]
		Adjacent	0.92 (0.26)	
Mmax% (%)	Involved	Injury site	1.41 (0.43)	*p* < 0.01 [0.51, 1.21]
		Adjacent	2.28 (0.50)	
	Contralateral	Mirrored injury site	2.49 (0.58)	*p* = 0.30 [− 0.49, 0.17]
		Adjacent	2.33 (0.67)	

^†^Data are presented as means (standard deviations)

^‡^*P*-values derived from separate two-sided, paired t-tests

*PSFR* peak spatial frequency radius, *SFA* spatial frequency analysis

A total of 9 athletes were included for analysis at RTS (Table 1). A total of 2 athletes were included for both TOI and RTS imaging. The mean number of days away from sport was 39.8 (23.7) days. The mean number of days between RTS and RTS US imaging was 3.4 (2.3) days. The median within-image difference of PSFR between the injured and healthy regions at TOI was 0.15 [0.03, 0.25] mm^−1^ (Table 3). The median within-image difference at TOI for Mmax% between the injury-matched and healthy portions was 0.71 [0.64, 0.98]%. At RTS, the corresponding within-image difference between the injury-matched locations and the healthy portions of the tissue were 0.05 [− 0.13, 0.07] mm^−1^ and 0.31 [− 0.22, 0.49]% for PSFR and Mmax%, respectively (Table 3). The within-image difference at RTS was lower than that at TOI for both PSFR (W = 79, *p* = 0.03) and Mmax% (W = 77, *p* = 0.04).

**Table 3:** Within-image difference of injured limb by time point for peak spatial frequency radius and Mmax%

	Time point relative to injury	Within-image difference^†^	*P*-value ^‡^ [95% Confidence Interval]
PSFR difference (mm^−1^)	Time of injury	0.15 [0.03, 0.25]	*p* = 0.03 [0.02, 0.31]
	Return to sport	0.05 [− 0.13, 0.07]	
Mmax% difference (%)	Time of injury	0.71 [0.64, 0.98]	*p* = 0.04 [0.16, 1.17]
	Return to sport	0.31 [− 0.22, 0.49]	

^†^Data are presented as median [interquartile range]. Within-image difference was calculated as $Healthy-Injury$

^‡^*P*-values derived from separate two-sided Wilcoxon rank sum tests

*PSFR* peak spatial frequency radius

## Discussion

This is the first study that has investigated changes in muscle tissue organization characterized by SFA following HSI. We did not observe differences in PSFR between the adjacent, healthy and injured regions in the same image at TOI. However, we did observe significantly lower Mmax% within the injured portion of the tissue relative to a healthy portion in the same image. There were no significant differences in PSFR and Mmax% in the contralateral limb between injury and adjacent-matched locations. Finally, we also detected significantly lower within-image differences in PSFR and Mmax% between the injured and adjacent tissue locations at RTS compared to TOI. Findings from this study provide a first step in determining the usefulness of SFA as a quantitative assessment of muscle injury.

### Aim 1: time of injury analysis of injured and contralateral limbs

Since SFA has not been performed in acutely injured muscle to the authors’ knowledge, it was previously unknown if SFA could detect differences between injured and non-injured tissue. Considering that degenerated tendons have a lower PSFR value compared to healthy tendons due to less compact fascicle bundles [15, 17, 26], it was hypothesized that PSFR would be lower in the injured region relative to the healthy portion of the tissue in response to structural disruption to the perimysium following HSI. The mean PSFR values were lower in the injured portion compared to the healthy region within the same image (Table 2) but did not strictly reach statistical significance (*p* = 0.06, 95% CI = [− 0.01, 0.25]). It is likely injured tissue results in a lower PSFR value, but this was not detected in the current study possibly due to the smaller sample size. Our findings may warrant further investigation with a larger sample size to determine if these trends persist.

Although PSFR has been the most commonly reported SFA parameter from the original subset [15], we also included Mmax% to further describe tissue organization. We chose this parameter due to minimal variation observed in pilot studies in both PSFR and Mmax% parameters when changing US machine settings. Therefore, we believe that PSFR and Mmax% are two parameters which are the most consistent from the original eight proposed SFA parameters [15] when comparing muscle structure with different image settings, over time, and in relationship to physiologically-meaningful tissue organization [18].

We observed a significant decrease in Mmax% in the injured regions of the tissue relative to the healthy, adjacent portion in the involved limb at TOI consistent with our initial hypothesis. The lower Mmax% value in the injured region is likely due to a combination of factors considering how this parameter is calculated, which encompasses both the prominence of the striated fascicular pattern and overall image brightness [15, 18, 20]. The prominence of the banded pattern is reduced in tissue where structural damage has occurred and the fascicular pattern has been disrupted. Additionally, the presence of edema increases image noise which is represented across all spatial frequencies and reduces the distinction of the overall light–dark repeating pattern observed in healthy tissue. Therefore, both the decreased prominence of the fascicular pattern, which was previously shown to be inversely related to Mmax% [18], and the increase in the noise floor suggest that the Mmax% parameter would decrease.

In the contralateral limb, mirrored injury and adjacent tissue locations served as a control condition to determine if differences in PSFR and Mmax% were present in healthy tissue. No differences in either PSFR or Mmax% were observed between the injury and adjacent-matched locations in the contralateral limb. Currently, SFA methods require that an ROI is drawn around the entire tendon or muscle thickness and then averaged across all kernels [15, 17–20, 26]. This value is taken as a representative measure of the entire tissue organization. Our non-significant findings within the different regions in the contralateral limb suggest that a single ROI across the entire muscle thickness may be representative of tissue organization within healthy tissue, which could reduce the time for quantitative analyses in future investigations of muscle injury. Despite the uncertainty that US machine settings may have on absolute SFA parameter extraction, it appears that in healthy muscle tissue between-limb comparisons may be performed when the same US machine and settings are used across the same individual bilaterally.

### Aim 2: within-image injured vs. healthy differences between TOI & RTS in injured limb

As a second aim, we investigated differences between regions of injury and healthy, adjacent tissue to compare within-image changes in PSFR and Mmax% over time. The median within-image absolute differences between the injured and healthy adjacent portions at TOI were 0.15 mm^−1^ for PSFR and 0.71% for Mmax%. These within-image differences decreased significantly at RTS to 0.05 mm^−1^ for PSFR and 0.31% for Mmax%. From our group’s previous investigation in the reliability of SFA in the hamstrings [20], we calculated a minimum detectable change (MDC) for PSFR as 0.19 mm^−1^ and 0.60% for Mmax% across the hamstrings. At RTS, the within-image difference for PSFR (median: 0.05 mm^−1^) and Mmax% (median: 0.31%) were close to zero and below the MDC of each respective parameter (Table 3), thereby confirming our hypothesis and suggesting that at RTS the tissue organization—as measured by SFA—resembled healthy tissue.

Future investigations may attempt to determine the sensitivity of SFA methods in identifying possible susceptible regions of re-injury and their relationship to muscle function. Given the high rate of injury recurrence, especially within the first 2 weeks of RTS [27, 28], the current assessments of muscle healing and readiness for RTS may be insufficient. SFA could provide more information into the overall tissue organization, perhaps as it pertains to susceptible areas of injury within and along the muscle [18, 29].

### Study limitations

There are some limitations to this study that should be noted. Not all US machine settings were constant between athletes or between limbs within the same individual as a musculoskeletal-trained sonographer manipulated parameters to optimize image acquisition in a clinically-relevant manner. Previous investigations have noted significant differences in quantitative US measures when different machines or settings are used [25] and it is currently unknown how manipulating typical machine settings used in musculoskeletal US influences the extracted SFA parameters. Therefore, we conducted a within-image analysis to minimize the effects machine settings may have on parameter extraction. We were also unable to determine within-subject change in PSFR and Mmax% as it relates to healing and recovery due to the cross-sectional design of this study. However, these results provide initial evidence in changes of SFA parameters between TOI and RTS, and future longitudinal studies should attempt to address this question. Finally, we did have a relatively small sample size included in this analysis. Future studies should include more subjects to determine if these trends persist.

## Conclusion

This work is the first to investigate how changes in muscle tissue organization are characterized by SFA parameters in B-mode US images following HSI and is an initial step in determining the usefulness of SFA for quantitative assessment of muscle injury and recovery. Although MRI is considered the ‘gold standard’ in musculoskeletal imaging, the practicality of MRI in clinical settings and/or large cohort studies is somewhat limited due to its high cost. Ultrasound is also well-suited for HSI diagnosis and may prove more feasible compared to MRI for both longitudinal assessments of healing and recovery and real-time tissue contraction dynamics. Future work will quantify longitudinal changes in SFA parameters throughout the recovery and rehabilitation process and in relation to conventional clinical measures of recovery. Using SFA methods in HSI applications—possibly in conjunction with assessment of the tissue material properties such as stiffness—may provide quantitative information of overall tissue integrity, which would aid in the RTS decision-making process in order to minimize time away from sport while also reducing re-injury risk.

## Data Availability

The images analyzed in the current study are not publicly available due to IRB restrictions. Data are available from the corresponding author upon reasonable request.

## References

[1] Pollock N, James SLJ, Lee JC, Chakraverty R (2014). British athletics muscle injury classification: a new grading system. Br J Sport Med.

[2] Valle X, Alentorn-Geli E, Tol JL, Hamilton B, Garrett WE, Pruna R (2017). Muscle injuries in sports: a new evidence-informed and expert consensus-based classification with clinical application. Sport Med.

[3] Mueller-Wohlfahrt H-W, Haensel L, Mithoefer K, Ekstrand J, English B, McNally S (2013). Terminology and classification of muscle injuries in sport: the Munich consensus statement. Br J Sports Med.

[4] Petersen J, Thorborg K, Nielsen MB, Skjødt T, Bolvig L, Bang N (2014). The diagnostic and prognostic value of ultrasonography in soccer players with acute hamstring injuries. Am J Sports Med.

[5] van Heumen M, Tol JL, de Vos R-J, Moen MH, Weir A, Orchard J (2017). The prognostic value of MRI in determining reinjury risk following acute hamstring injury: a systematic review. Br J Sports Med.

[6] Connell DA, Schneider-Kolsky ME, Hoving JL, Malara F, Buchbinder R, Koulouris G (2004). Longitudinal study comparing sonographic and MRI assessments of acute and healing hamstring injuries. Am J Roentgenol.

[7] Whittaker JL, Ellis R, Hodges PW, Osullivan C, Hides J, Fernandez-Carnero S (2019). Imaging with ultrasound in physical therapy: What is the PT’s scope of practice? A competency-based educational model and training recommendations. Br J Sports Med.

[8] Lee JC, Healy J (2004). Sonography of Lower Limb Muscle Injury. Am J Roentgenol.

[9] Crema MD, Yamada AF, Guermazi A, Roemer FW, Skaf AY (2015). Imaging techniques for muscle injury in sports medicine and clinical relevance. Curr Rev Musculoskelet Med.

[10] Chopp-Hurley JN, Wiebenga EG, Bulbrook BD, Keir PJ, Maly MR (2020). Evaluating the relationship between quadriceps muscle quality captured using ultrasound with clinical severity in women with knee osteoarthritis. Clin Biomech.

[11] Watanabe Y, Yamada Y, Fukumoto Y, Ishihara T, Yokoyama K, Yoshida T (2013). Echo intensity obtained from ultrasonography images reflecting muscle strength in elderly men. Clin Interv Aging.

[12] Ismail C, Zabal J, Hernandez HJ, Woletz P, Manning H, Teixeira C (2015). Diagnostic ultrasound estimates of muscle mass and muscle quality discriminate between women with and without sarcopenia. Front Physiol..

[13] Fukumoto Y, Ikezoe T, Yamada Y, Tsukagoshi R, Nakamura M, Mori N (2012). Skeletal muscle quality assessed from echo intensity is associated with muscle strength of middle-aged and elderly persons. Eur J Appl Physiol.

[14] Strasser EM, Draskovits T, Praschak M, Quittan M, Graf A (2013). Association between ultrasound measurements of muscle thickness, pennation angle, echogenicity and skeletal muscle strength in the elderly. Age.

[15] Bashford GR, Tomsen N, Arya S, Burnfield JM, Kulig K (2008). Tendinopathy discrimination by use of spatial frequency parameters in ultrasound B-mode images. IEEE Trans Med Imaging.

[16] Kulig K, Chang Y-J, Winiarski S, Bashford GR (2016). Ultrasound-based tendon micromorphology predicts mechanical characteristics of degenerated tendons. Ultrasound Med Biol.

[17] Kulig K, Landel R, Chang YJ, Hannanvash N, Reischl SF, Song P (2013). Patellar tendon morphology in volleyball athletes with and without patellar tendinopathy. Scand J Med Sci Sport.

[18] Crawford SK, Lee KS, Bashford GR, Heiderscheit BC (2021). Spatial-frequency analysis of the anatomical differences in hamstring muscles. Ultrason Imaging.

[19] Cassel M, Risch L, Mayer F, Kaplick H, Engel A, Kulig K (2019). Achilles tendon morphology assessed using image based spatial frequency analysis is altered among healthy elite adolescent athletes compared to recreationally active controls. J Sci Med Sport.

[20] Crawford SK, Lee KS, Bashford GR, Heiderscheit BC (2020). Intra-session and inter-rater reliability of spatial frequency analysis methods in skeletal muscle. PLoS ONE.

[21] Wangensteen A, Bahr R, Van Linschoten R, Almusa E, Whiteley R, Witvrouw E (2017). MRI appearance does not change in the first 7 days after acute hamstring injury—a prospective study. Br J Sports Med.

[22] Heiderscheit BC, Sherry MA, Silder A, Chumanov ES, Thelen DG (2010). Hamstring strain injuries: recommendations for diagnosis, rehabilitation, and injury prevention. J Orthop Sports Phys Ther.

[23] Gielen JL, Robinson P, Van Dyck P, Van der Stappen A, Vanhoenacker FM. Muscle Injuries. In: Imaging of Orthopedic Sports Injuries. Berlin: Springer; 2007. p. 15–39.

[24] Takebayashi S, Takasawa H, Banzai Y, Miki H, Sasaki R, Itoh Y (1995). Sonographic findings in muscle strain injury: clinical and MR imaging correlation. J Ultrasound Med.

[25] Zaidman CM, Holland MR, Hughes MS (2012). Quantitative ultrasound of skeletal muscle: reliable measurements of calibrated muscle backscatter from different ultrasound systems. Ultrasound Med Biol.

[26] Ho K-Y, Baquet A, Chang Y-J, Chien L-C, Harty M, Bashford G (2019). Factors related to intra-tendinous morphology of Achilles tendon in runners. PLoS ONE.

[27] Malliaropoulos N, Papacostas E, Kiritsi O, Papalada A, Gougoulias N, Maffulli N (2010). Posterior thigh muscle injuries in elite track and field athletes. Am J Sports Med.

[28] De Vos RJ, Reurink G, Goudswaard GJ, Moen MH, Weir A, Tol JL (2014). Clinical findings just after return to play predict hamstring re-injury, but baseline MRI findings do not. Br J Sports Med.

[29] De Smet AA, Best TM (2000). MR imaging of the distribution and location of acute hamstring injuries in athletes. Am J Roentgenol.

